# Morphological Change in Optical Coherence Tomography and Functional Outcomes in Epiretinal Membrane Peeling with or without SF6 Tamponade

**DOI:** 10.3390/diagnostics14141483

**Published:** 2024-07-11

**Authors:** Yi-Chun Chi, Wei-Lun Chu, Kuo-Jen Chen, Kai-Chun Cheng

**Affiliations:** 1 Department of Ophthalmology, Kaohsiung Medical University Hospital, Kaohsiung Medical University, Kaohsiung 807, Taiwan; jenniferchi1308@gmail.com; 2 Department of General Medicine, Kaohsiung Medical University Hospital, Kaohsiung 807, Taiwan; t5368836@gmail.com; 3 Department of Ophthalmology, Kaohsiung Municipal Siaogang Hospital, Kaohsiung 812, Taiwan; 0870649@kmuh.org.tw; 4 Department of Ophthalmology, School of Medicine, College of Medicine, Kaohsiung Medical University, Kaohsiung 807, Taiwan

**Keywords:** epiretinal membrane peeling, pars plana vitrectomy, sulfur hexafluoride (SF6), visual outcome, macular thickness

## Abstract

Background: The present study compares the anatomical and functional outcomes (best-corrected visual acuity (BCVA) and central macular thickness (CMT)) among membrane peeling with or without SF6 tamponade in patients with epiretinal membrane. Methods: We retrospectively reviewed patients diagnosed with macular pucker who underwent pars plana vitrectomy and membrane peeling in a tertiary center in Taiwan from January 2021 to December 2022. Subjects were categorized into with or without SF6 tamponade groups (SF6 group and BSS group). Postoperative intraocular pressure and complications were documented. Logistic regression analyses were performed to identify the prognostic factors during follow-up. Results: A total of 89 eyes were enrolled, including 34 eyes in the BSS group and 55 eyes in the SF6 group. The mean age was 66 years old, and a female predilection was demonstrated. Both groups possessed statistically significant improvement in BCVA and CMT after the operation. There was no significant difference in CMT between the groups at any time of observation, yet we observed significant differences in baseline BCVA and BCVA at last follow-up among the two groups. Both groups yielded an approximate enhancement of LogMAR 0.3 in BCVA postoperatively. There was no significant difference noted in postoperative IOP between the two groups. Conclusion: Membrane peeling with or without SF6 tamponade yields comparable outcomes anatomically and functionally. This may indicate that SF6 tamponade for idiopathic macular pucker surgery may not provide extra benefit, and therefore warrants reconsideration as standard procedure.

## 1. Introduction

Epiretinal membrane (ERM), first documented in 1865, manifests as an avascular, fibrocellular layer that proliferates on the inner surface of the retina. Known by various names such as primary retinal folds, wrinkling of the inner retinal surface, preretinal macular fibrosis (PMF) or gliosis, cellophane maculopathy, and macular pucker [1,2]. This condition is chiefly associated with advancing age, with most cases occurring after the age of 50 and peaking in the 7th decade [3,4,5]. Additionally, studies suggest ethnicity-specific prevalence rates, with individuals of Chinese descent reportedly at a higher risk of developing ERMs [6,7].

ERMs may be idiopathic or secondary. In idiopathic ERMs (iERMs), cell proliferation follows posterior vitreous detachment (PVD) and a break in the ILM [8]. Secondary ERMs arise from coexisting or preceding ocular diseases, with common causes including previous cataract surgery, diabetic retinopathy, trauma, and retinal vein occlusion [3,9].

ERMs typically consist of two layers overlying the internal limiting membrane (ILM). The outer layer comprises non-cellular extracellular matrix (ECM) proteins with bundles of randomly oriented fibrils, while the inner layer encompasses epiretinal cell layers derived from various cell types including glial cells, retinal pigment epithelium (RPE), fibrocytes, myofibroblasts, fibroastrocytes, laminocytes, hyalocytes, macrophages, or fibroblasts [2,10].

While ERMs can be asymptomatic initially, progressive fibrocellular contraction can lead to irregular wrinkling of the inner retinal layer, affecting vision, particularly in the macular or peri-macular region. Symptoms may include blurred vision, metamorphopsia, loss of stereopsis, and aniseikonia.

For patients experiencing symptomatic visual disturbances, timely surgical intervention with pars plana vitrectomy (PPV) and ERM peeling is recommended to restore the foveal structure and to prevent visual disturbances worsening [11,12]. The removal of the ILM, which acts as a scaffold for cellular proliferation, has become common practice alongside ERM peeling to reduce the likelihood of recurrence, despite its uncertain impact on VA outcomes [13,14,15]. During surgery, gas–fluid exchange may be performed after membrane peeling to flatten the retina and reduce retinal folds. For the management of iERM, gas tamponade with air or sulfur hexafluoride (SF6) is occasionally employed [16], whereas perfluoropropane (C3F8), a long-acting gas, is typically reserved for more complicated cases involving vitreomacular traction or macular hole [17,18].

The debate on whether to perform gas tamponade in iERM is ongoing [19,20]. This study aims to compare surgical outcomes, including central macular thickness (CMT) and best-corrected visual acuity (BCVA) in patients undergoing PPV with and without gas tamponade for idiopathic epiretinal membrane (iERM). The postoperative intraocular pressure (IOP) and other complications among the two groups were also documented.

## 2. Materials and Methods

We conducted a retrospective analysis of patients diagnosed with ERM who underwent pars plana vitrectomy (PPVT) and full ILM/ERM peeling with or without gas tamponade, at Kaohsiung Medical University Hospital, a tertiary center in Taiwan, from January 2021 to December 2022. ERM was diagnosed via spectral-domain optical coherence tomography (SD-OCT) (Spectralis, Heidelberg, Germany). We excluded individuals with (1) postoperative follow-up duration less than 6 months, (2) ERM concomitant with lamellar hole, vitreomacular traction (VMT), retinoschisis, macular hole (MH) or macular scar, (3) severe non-proliferative diabetic retinopathy (NPDR) or proliferative diabetic retinopathy (PDR), (4) history of previous vitreoretinal surgery, such as vitrectomy for rhegmatogenous retinal detachment (RRD) and MH, (5) cytomegalovirus (CMV) retinitis, underlying neovascular age-related macular degeneration (nAMD) receiving intravitreal injection (IVI) treatment, (6) simultaneous endolaser treatment, IVI of anti-VEGF, or IVI of Ozurdex^®^ (dexamethasone intravitreal implant) during vitrectomy surgery for ERM, and (7) advanced, severe, or end-stage glaucoma staging based on the Humphry visual field [21]. This study was approved by the institutional review board of Kaohsiung Medical University Hospital (KMUHIRB-E(I)-20240163) under the ethical standards of the Declaration of Helsinki. Informed consent was waived due to the retrospective nature of the study.

The enrolled subjects were divided into two groups: those receiving PPVT and full ILM/ERM peeling without gas tamponade, and those receiving PPVT with full ILM/ERM peeling plus gas tamponade. All of the gas applied in the current study was sulfur hexafluoride (SF6). Whether the patient received surgery with or without gas was determined by the surgeon’s preference of the patient’s consent after thorough shared decision-making discussions. All surgeons were highly trained and possessed over 5 years of experience in vitreoretinal surgery.

### 2.1. Surgical Techniques

The PPVT was performed through 3 ports of 23- or 25-gauge scleral tunnel. Triamcinolone was used for visibility of residual hyaloid to assist PPVT in some cases. After vitrectomy, indocyanine green (ICG) dye was applied for staining at the posterior pole for one minute. ERM and ILM were peeled off carefully with forceps mostly via a centripetal manner. Combination surgery with phacoemulsification and posterior chamber intraocular lens implantation (PCIOL) was performed if the lens opacity interfered with the clarity of the fundus. In the cases of PPVT and membrane peeling only, balanced salt solution (BSS) was left in the vitreous cavity; in the cases of PPVT and membrane peeling with gas tamponade, full-filled SF6 tamponade was performed lastly. Patients with gas tamponade were advised to maintain a prone position postoperatively.

### 2.2. Outcome Measurements

Demographic data of age, gender, eye laterality, axial length (AL), underlying medical conditions, whether glaucoma, and preoperative lens status (whether phakic or pseudophakic) were recorded. The outcomes of interest were the BCVA and CMT. We collected information on preoperative BCVA and CMT, as well as those at postoperative intervals of 1 month, 3 months, 6 months and at the final follow-up. The BCVA using semi-quantitative scale “counting fingers” was converted to decimal acuity of 0.014 (corresponding to logMAR = 1.85), and hand motion (HM) was replaced by decimal acuity of 0.005 (corresponding to logMAR = 2.30) [22]. Moreover, we documented the preoperative IOP, and IOP at one week as well as one month postoperatively.

### 2.3. Statistical Analysis

The statistical analyses were performed with Statistical Product and Service Solutions (SPSS), version 20. Data were presented with mean and standard deviation (SD) in continuous variables while with numbers and proportion (%) in categorical variables. The comparison between the two groups was conducted with Mann–Whitney U test, chi-square test, and Fisher exact test, while comparison between baseline and last follow-up was conducted with Wilcoxon signed-rank test. Logistic regression analyses were applied for identifying the predictors of a worse postoperative best-corrected visual acuity, LogMAR > 0.23, at last follow-up. A *p* value less than 0.05 was defined as statistically significant.

## 3. Results

Overall, 277 eyes with macular pucker which underwent PPVT were reviewed. We excluded 41 eyes due to a postoperative follow-up duration less than 6 months; 45 eyes concomitant with lamellar hole, VMT, retinoschisis, MH, or macular scar; 23 eyes with severe NPDR or PDR; nine with previous PPVT for RRD or MH; six nAMD under IVI therapy; one CMV retinitis; eight advanced glaucoma; 50 eyes received endolaser treatment or IVI of Ozurdex^®^ (Allergan, Irvine, CA, USA) during macular pucker surgery.

A total of 97 eyes were enrolled in the current study. Among them, six patients suffered from complications following surgery and were excluded from the final analysis. Complications included one eye with postoperative RRD, two eyes with recurrent macular pucker treated with another membrane peeling surgery, one recurrent macular edema treated with IVI of Ozurdex^®^, one waxing and waning uveitis postoperatively, and one central visual field defect with macular scarring after operation. Eventually, 89 eyes were included and analyzed.

The demographic characteristics were shown in Table 1. A total of 34 eyes underwent PPVT and membrane peeling without gas tamponade, categorized in the BSS group, while 55 eyes underwent PPVT and membrane peeling with gas tamponade, categorized in the SF6 group. The mean age was 64.44 ± 9.11 years old in the BSS group, and 66.62 ± 6.09 years old in the SF6 group. A slight female predominance was demonstrated in our cohort (53% female in the BSS group and 67% in the SF6 group). The top underlying comorbidities included hypertension, diabetes mellitus (DM), hyperlipidemia, and thyroid disease. Most patients were phakic at the time of receiving macular pucker surgery. Fourteen cases in the BSS group received combined surgery with phacoemulsification and PCIOL, and 49 cases in the SF6 group received the combined surgery. Between the two groups, there were no significant differences in age (*p* = 0.209), gender (*p* = 0.176), laterality (*p* = 0.676), preoperative lens status (*p* = 0.072), axial length (*p* = 0.219), follow-up duration (*p* = 0.603), baseline IOP (*p* = 0.438), and baseline CMT (*p* = 0.497); nevertheless, a significant difference was found in baseline BCVA (*p* = 0.004).

Table 2 reveals the postoperative outcomes. There were significant differences in the CMT and BCVA at the last follow-up compared to the baseline in either BSS or SF6 group (*p* < 0.01), which indicated the significant decrease in CMT and improvement in BCVA after surgery. There was no significant difference in CMT between BSS and SF6 group at any time of measurement (preoperative baseline, postoperative 1 month, postoperative 3 months, postoperative 6 months, and last follow-up). Significant differences were observed in BCVA between BSS and SF6 at baseline (*p* = 0.04), postoperative 1 month (*p* = 0.012), and last follow-up (*p* = 0.003). The average of BCVA (logMAR) improved by approximately 0.3 in both the BSS (from 0.53 to 0.21) and the SF6 group (from 0.78 to 0.46).

To measure the potential postoperative IOP spikes in the use of SF6, we compared IOP in the BSS and the SF6 group (Table 3). No significant differences were noted in-between the two groups at preoperative baseline (*p* = 0.438), postoperative 1 week (*p* = 0.196), and postoperative 1 month (*p* = 0.387).

Logistic regression analyses were performed to identify the predictors of a worse postoperative BCVA (Table 4). In the univariate model, old age, short axial length, SF6 tamponade, and a worse BCVA were significant predictors of a poorer visual outcome. However, only a short axial length and a worse preoperative BCVA achieved statistical significance in the multivariate analysis.

## 4. Discussion

ERM is a relatively common cause of visual impairment in the elderly. The prevalence largely varies from 3.4% [23] to 39.0% [7] in the Chinese population. Xiao et al. conducted a meta-analysis and demonstrated a 9.1% age-standardized prevalence of any type of ERM [3]. Greater age, female sex, myopia, DM, hyperlipidemia, and cigarette smoking were common risk factors discussed [1,3,7,23,24]. In our cohort, we observed an advanced age with an average of 65.79 years old and female predilection constituted up to 62%, which echoed previous studies [3,7]. The prevalence of DM (17%) was less than that of previously reported [7]. This could be attributed to the exclusion of severe NPDR and PDR, which were frequently characterized with retinal fibrosis and macular edema, in order to minimize the potential interference with the outcomes in the current study. Hyperlipidemia, thyroid disease, and end-stage renal disease (ESRD) on hemodialysis were detected in a notable proportion of cases; nevertheless, these underlying comorbidities did not exceed the prevalence of the general population in Taiwan [25,26,27].

The diagnosis of ERM is often made clinically and is straightforward. Fundoscopy or color photos may show an abnormal reflectivity with a glistening, translucent membrane over the macula. Preretinal fibrosis, retinal folds secondary to contractions, may present when the cellophane membrane thickens. With the advancement of image resolution, OCT has become the gold standard for diagnosing ERM and evaluating the microstructure, such as disruption of the ellipsoid zone and the morphology of the inner retinal layers. Govetto et al. proposed a novel staging system based on the morphology upon OCT: stage 1 was defined as thin ERM with a preserved foveal depression; stage 2 was characterized by widening of the outer nuclear layer and loss of the foveal depression; stage 3 was identified by the presence of continuous ectopic inner foveal layers crossing the entire foveal area; stage 4 indicated the addition of remarkable anatomic disruption of the macula [28]. All of our subjects enrolled were characterized within stage 2 and 3.

ERM is considered as a wound-healing process which leads to contractile, fibrovascular proliferation over the vitreoretinal interface. Aging and PVD were the pathogenic factors in iERM formation. The epidemiologic studies showed that iERM was associated with PVD in over 70% of cases at the time of diagnosis [2,29,30]. The pathogenesis of iERM is still being investigated. The classic explanation was proposed by Foos, who hypothesized that the PVD could lead to defects in the ILM and allow retinal glial cells (Müller cells and astrocytes) to migrate outward over the inner retinal surface [31]. However, subsequent immunohistochemical (IHC) investigations indicate that ILM pores are a rare finding [32]. Another pathway is proposed, that the hyalocytes residing in the cortical vitreous remnants on the ILM can be activated by growth factors, such as basic fibroblast growth factor (bFGF), transforming growth factor (TGF)-β, and nerve growth factor (NFG) [8,33], and result in cellular proliferation and myofibroblast differentiation, leading to iERM formation and contraction. Modification of the extracellular matrix (ECM), such as advanced glycation end products (AGEs) and collagens accumulation, also plays a role in iERM formation. They may increase the rigidity of vitreoretinal collagens and promote the fibrotic process by inducing the myofibroblast transdifferentiation and fibroblast proliferation [2].

With the understanding of the ERM histology, some studies attempted to link the association between clinical characteristics and histopathology of iERM. Ngan et al. conducted a cross-sectional study of 35 iERMs at stage 1 or 2 (Gass 1993) [34], and identified six different cell types in HE staining, including glial cell, fibroblast, myofibroblast, macrophage, lymphocyte, and neutrophil. They concluded that the number of cell types significantly correlated to CMT and symptom duration [35]. Recently, a novel tissue submission procedure was introduced and enabled clinicians to obtain high-quality images for IHC analysis [36]. Wang et al. reported that more glial cells and myofibroblasts in ERMs than ILMs, and more macrophage-like cells and RPE cells in secondary ERMs than iERMs [36]. The same research team demonstrated correlations between clinical and histopathological characteristics in iERM in 2022 [37]. The main conclusion revealed that the glial cell density and high cellularity may result in poor postoperative BCVA and BCVA improvement. Moreover, several studies had reported a dissociation between central foveal thickness and postoperative visual acuity [38,39]. This may imply the multifaceted causes influencing visual acuity in ERM, not solely reliant on CMT, as evidenced by the occasional dissociation of CMT and BCVA in our study.

All subjects involved in the current study underwent double peeling, which included the removal of both ERM and ILM. Although ILM peeling in ERM surgeries has largely been applied in clinical practice, there is still a lack of consensus regarding its benefits. It is believed that ILM serves as a scaffold for cellular proliferation, and the peeling of ILM ensures a more complete removal of ERM, thereby reducing ERM recurrence and the need for reoperation [13,15]. However, peeling of the ILM, the basal lamina representing the footplate of Müller cells, may impair these cells and result in glial apoptosis. It may lead to greater micro-scotomas and inner retinal dimpling, which further impair vision [14,40]. Furthermore, the application of dyes to enhance the visibility of the ILM during surgery could potentially impair retinal function [41]. Various dyes were utilized in membrane peeling, including ICG, trypan blue, and brilliant blue (BBG). All surgeries performed in the present study were 0.5% ICG assisted. ICG may result in delayed RPE and glial cell damage with 1% ICG [42], and increases the light induced oxidative stress in RPE cells [42,43]. Despite the relatively safe application of 0.5% ICG reported by Jackson et al. [42], the potential hazards should not be overlooked.

In the current study, we assessed the impact of membrane peeling with or without SF6 tamponade for iERM. We monitored the changes in BCVA and CMT over a six-month postoperative period. Our findings revealed that BCVA and CMT improved gradually in both groups, with no significant differences in CMT between those who received SF6 tamponade and those filled with BSS. However, there were significant differences in the BCVA between the two groups at baseline, postoperative 1 month, and last follow-up. This may imply that the worse the vision, the more prone patients and surgeons were to select the operation with gas tamponade. Moreover, the better the preoperative vision, the better the vision at final postoperative follow-up. Nevertheless, at the last follow-up, both groups showed an improvement in BCVA compared to preoperative levels, with an approximate enhancement of 0.3 logMAR, which was comparable to that reported by Leitritz et al. [20].

Whether to perform gas tamponade and which type of gas is superior in iERM surgery remain subjects of ongoing debate. A pilot study conducted by Emrani et al. in 2014 suggested significant improvements in morphology and function in eyes with gas tamponade (air, SF6 or perfluoropropane (C3F8)) compared to those without gas tamponade in ERM surgeries [44]. However, further studies failed to replicate the finding of the significant superiority of the functional outcome, and instead suggested that there is no significant difference in BCVA between ERM surgeries with or without the utilization of gas. Leitritz et al. compared the surgical outcomes, including visual acuity and foveal contour changes, in epiretinal membrane surgery using either air tamponade or BSS, and reported comparable results in BCVA among both groups but a significantly better contour in the air tamponade group [20]. Jang et al. conducted another comparative analysis between the use of air tamponade and no tamponade after iERM surgery. They concluded that there was no difference in postoperative visual acuity, CMT, and peripapillary nerve fiber layer thickness between the two cohorts [19]. Chabot undertook a prospective trial to compare the impact of 20% SF6 versus air tamponade in ERM surgery. Similarly, they stated that no significant differences were observed in BCVA and central retinal thickness [16]. This study contributes to evidence that the application of SF6 in ERM surgery yields similar morphologic outcomes in terms of improving CMT compared to surgeries performed without gas tamponade.

Predictors of final visual outcomes following membrane peeling surgery were analyzed with logistic regression in Table 4. Age, AL, whether SF6 tamponade was utilized, and preoperative BCVA were significant predictors of postoperative BCVA in univariate analysis. However, when it comes to the multivariate model, only AL and preoperative BCVA significantly predicted the final BCVA, which revealed that a shorter AL and a worse preoperative BCVA might indicate a worse postoperative BCVA. Previous studies have shown that preoperative vision significantly predicts postoperative functional outcome [38,39,45]. A better pretreatment BCVA may indicate a lower level of irreversible retinal damage or pathological changes, as supported by Inoue et al. that a disrupted preoperative inner segment/outer segment junction integrity is in relation to worse preoperative BCVA and postoperative vision [45]. A shorter AL leading to poorer visual outcomes might be the result of the tractional forces at the vitreoretinal interface [46]. Eyes with a shorter AL may lack posterior vitreous detachment (PVD) or experience a macula-sparing PVD, which chronically induces tractional forces to the macula. Additionally, iERM is influenced by the migration of Müller glial cells and cytokines, resulting in enhanced adhesion to the retina and greater tractional force in a shorter AL eye [46]. Procedures to induce PVD and remove ERM in a shorter AL eye would apply a more intense tractional force to the macula, and consequently damage the neural retina intraoperatively, resulting in a worse postoperative functional outcome. In contrast, eyes with a relatively longer AL may more readily develop PVD or vitreoschisis due to vitreous liquefaction, exerting less traction on the retina and causing fewer modifications to the ERM, thereby minimizing macular damage.

Complications related to macular peeling are observed in other vitreoretinal procedures, often more attributed to PPVT rather than the peeling maneuvers themselves. These include cataract progression, increases in IOP, retinal tears with or without consequent retinal detachment, visual field defects, vitreous hemorrhage, ocular hypotony, dislocation of the intraocular lens, macular phototoxicity, RPE changes, and endophthalmitis [16,47,48,49]. Additionally, certain complications are directly linked to macular peeling, such as focal retinal hemorrhages and edema, which typically resolve spontaneously. There are also occasional reports of retinoschisis and macular edema following macular peeling [16,48]. In the current study, we reported one eye with postoperative RRD, two eyes with recurrent ERM receiving subsequent membrane peeling, one recurrent macular edema treated with IVI of dexamethasone, one with postoperative ocular inflammation requiring further treatment, and one with a central visual field defect with macular scarring. Certain iatrogenic retinal breaks and retinal detachments might have been overlooked, as we intentionally omitted intraoperative endolaser treatment to maintain a standardized procedure.

IOP was the secondary outcome measure in the present study for evaluating the potential complication of ocular hypertension particularly in gas-filled eyes. PPVT has been proposed as a contributing factor of secondary open-angle glaucoma [50,51]. Previous studies have demonstrated that a simple PPVT can lead to significant elevation in IOP, with reported estimated incidence rates ranging from 15% to 20% [50,51,52]. It is hypothesized that elevated IOP following PPVT may be due to the increased oxygen diffusion from the vitreous cavity to the anterior chamber [52], triggering oxidative stress within the trabecular meshwork, thereby contributing to the rise in IOP [52,53]. The incidence of elevated IOP tends to be even greater when PPVT is combined with intravitreal gas tamponade, ranging widely from 6% to 67% in SF6 and 18% to 69% in C3F8 [54,55,56]. The expansile gas expands rapidly within the vitreous cavity, causing ciliary body edema and iridocorneal apposition, consequently leading to secondary angle closure, regardless of the presence of pupillary block [54]. The clinical longevity of a gas bubble spans from 13 to 24 days for 30% SF6, 28 to 44 days for 20% hexafluoroethane (C2F6), and 59 to 79 days for 15% C3F8 [57]. As a result of utilizing SF6 in our cohort, we monitored IOP within the first month postoperatively. Twelve patients experienced an increase in IOP of at least 4 mmHg, exceeding 21 mmHg (24.6 ± 2.5 mmHg) at the first week post-surgery. Notably, 10 out of these 12 patients underwent membrane peeling with SF6 gas tamponade. By one month postoperatively, the IOP of these 12 patients reduced to 16.9 ± 4.7 mmHg, and only two individuals in the gas tamponade group remained with an IOP greater than 21 mmHg. However, our results demonstrated no statistically significant difference in IOP between the gas and BSS tamponade groups at any time point.

There are several limitations in the current study. The major one is the limitation deriving from the retrospective manner. Although we established relatively strict criteria to identify iERM and the surgery, the absence of randomization could lead to baseline differences in disease severity between the groups. Retrospective studies rely largely on the accuracy of medical records, inevitably leading to the challenge of missing data. It is also difficult to obtain tissue for histologic examinations or pathogenesis investigations in a retrospective manner. Moreover, there is a relatively small sample size in the current study that might amplify the statistical bias. Furthermore, the design involving multiple surgeons might introduce variability and bias stemming from individual preference and technique, even though only experienced vitrectomy surgeons participated in the current study. Nevertheless, to our knowledge, this is the first study to compare the effect of SF6 versus no gas tamponade after vitrectomy for iERM and evaluate the predictors of ultimate functional outcomes in ERM surgery.

## 5. Conclusions

Conclusively, we illustrated comparable anatomical and functional outcomes among membrane peeling surgery with or without SF6 tamponade for iERM. A shorter AL and poorer preoperative BCVA significantly correlate with worse BCVA at the final follow-up. There were no significant elevated postoperative IOP as well as complications between the two groups. This study may indicate that SF6 tamponade for iERM surgery may not provide extra benefits, and therefore warrants reconsideration as a standard procedure.

## Figures and Tables

**Table 1:** Demographic characteristics of eyes with macular pucker receiving membrane peeling surgery.

	Total (N = 89)	BSS (N = 34)	SF6 (N = 55)	*p* Value
Age (years)	65.79 ± 7.47	64.44 ± 9.11	66.62 ± 6.09	0.209 ^a^
Gender				0.176 ^b^
Male	34 (38%)	16 (47%)	18 (33%)	
Female	55 (62%)	18 (53%)	37 (67%)	
Comorbidities				
Hypertension	42 (47%)	19 (56%)	23 (42%)	
Diabetes mellitus	15 (17%)	8 (24%)	7 (13%)	
Hyperlipidemia	15 (17%)	3 (9%)	12 (22%)	
Thyroid disease	7 (8%)	3 (9%)	4 (7%)	
Status post retinal laser	6 (7%)	4 (12%)	1 (2%)	
ESRD on hemodialysis	4 (4%)	3 (9%)	1 (2%)	
Laterality				0.676 ^b^
OD	47 (53%)	17 (50%)	30 (55%)	
OS	42 (47%)	17 (50%)	25 (45%)	
Lens status				0.072 ^c^
Phakic	76 (85%)	26 (76%)	50 (91%)	
Pseudophakic	13 (25%)	8 (24%)	5 (9%)	
Axial length (mm)	24.68 ± 1.95	24.83 ± 1.80	24.59 ± 2.03	0.219 ^a^
Follow-up duration (months)	18.20 ± 8.57	17.62 ± 8.59	18.56 ± 8.54	0.603 ^a^
Baseline IOP (mmHg)	15.25 ± 3.29	14.92 ± 3.12	15.45 ± 3.37	0.438 ^a^
Baseline BCVA (logMAR)	0.68 ± 0.36	0.53 ± 0.28	0.78 ± 0.37	0.004 ^a^*
Baseline CMT (µm)	465.46 ± 103.10	460 ± 100.55	468.65 ± 104.53	0.497 ^a^

BSS = balanced salt solution; SF6 = sulfur hexafluoride; OD = right eye; OS = left eye; IOP = intraocular pressure; BCVA = best-corrected visual acuity; CMT = central macular thickness; ESRD = end-stage renal disease; * indicated statistically significant; ^a^ Mann–Whitney U; ^b^ chi-square; ^c^ Fisher exact test (double tailed).

**Table 2:** Comparison of the outcomes (central macular thickness and best-corrected visual acuity) among membrane peeling surgery with or without gas tamponade.

	CMT (µm)		BCVA (logMAR)			
	BSS (N = 34)	SF6 (N = 55)	*p* Value	BSS (N = 34)	SF6 (N = 55)	*p* Value
Baseline	460 ± 100.55	468.65 ± 104.53	0.497 ^a^	0.53 ± 0.28	0.78 ± 0.37	0.004 ^a^*
Postoperative 1 month	400.82 ± 69.72	407.91 ± 66.57	0.639 ^a^	0.43 ± 0.26	0.71 ± 0.49	0.012 ^a^*
Postoperative 3 months	376.94 ± 61.91	390.40 ± 54.58	0.328 ^a^	0.43 ± 0.35	0.61 ± 0.41	0.055 ^a^
Postoperative 6 months	367.18 ± 59.61	375.63 ± 52.07	0.461 ^a^	0.48 ± 0.47	0.51 ± 0.42	0.511 ^a^
Last follow-up	365.73 ± 57.17	373.51 ± 63.13	0.924 ^a^	0.21 ± 0.26	0.46 ± 0.47	0.003 ^a^*
*p* value	<0.001 ^b^*	<0.001 ^b^*		<0.001 ^b^*	<0.001 ^b^*	

BCVA = best-corrected visual acuity; CMT = central macular thickness; BSS = balanced salt solution; SF6 = sulfur hexafluoride; * indicated statistically significant; ^a^ Mann–Whitney U; ^b^ Wilcoxon signed-rank test.

**Table 3:** Comparison of intraocular pressure among membrane peeling surgery with or without gas tamponade.

IOP	Baseline	Postoperative 1 Week	Postoperative 1 Month
BSS (N = 34)	14.92 ± 3.12	13.90 ± 4.17	15.28 ± 4.96
SF6 (N = 55)	15.45 ± 3.37	15.64 ± 5.55	14.36 ± 3.94
*p* value	0.438 ^a^	0.196 ^a^	0.387 ^a^

IOP = intraocular pressure; BSS = balanced salt solution; SF6 = sulfur hexafluoride; ^a^ Mann–Whitney U.

**Table 4:** Logistic regression analyses of predictors of worse postoperative best-corrected visual acuity at last follow-up (LogMAR > 0.23).

	Univariate Analyses	Multivariate Analyses		
	Odds Ratio (95% CI)	*p* Value	Odds Ratio (95% CI)	*p* Value
Age	1.143 (1.062–1.231)	<0.001 *	1.187 (0.991–1.422)	0.063
Gender (Reference: female)	1.085 (0.461–2.554)	0.852	0.593 (0.066–5.293)	0.640
PPVT alone or combined cataract surgery (Reference: PPVT alone)	0.452 (0.175–1.165)	0.100	7.829 (0.423–144.795)	0.167
Axial length	0.619 (0.434–0.883)	0.008 *	0.152 (0.039–0.600)	0.007 *
BSS or SF6 (Reference: SF6)	0.176 (0.067–0.461)	<0.001 *	0.139 (0.017–1.145)	0.067
Preoperative BCVA (logMAR)	15.625 (3.387–72.086)	<0.001 *	192.342 (2.316–15,971.393)	0.020 *
Preoperative CMT	1.001 (0.997–1.005)	0.619	1.006 (0.989–1.022)	0.498
Last CMT	1.002 (0.995–1.010)	0.513	0.990 (0.964–1.017)	0.450

CI = confidence interval; BCVA = best-corrected visual acuity; CMT = central macular thickness; PPVT = pars plana vitrectomy; BSS = balanced salt solution; SF6 = sulfur hexafluoride; * indicated statistically significant.

## Data Availability

The data presented in this study are available on request from the corresponding author. The data are not publicly available due to privacy.
